# Functional and educational outcomes after treatment for intracranial arteriovenous malformations in children

**DOI:** 10.1007/s00701-018-3665-y

**Published:** 2018-09-07

**Authors:** Max J. van Essen, Kuo Sen Han, Rob T. H. Lo, Peter Woerdeman, Albert van der Zwan, Tristan P. C. van Doormaal

**Affiliations:** 1 Department of Neurosurgery, University Medical Center Utrecht, Heidelberglaan 100, 3584 CX Utrecht, The Netherlands; 2 Department of Radiology, University Medical Center Utrecht, Heidelberglaan, 100 Utrecht, The Netherlands; 3 Brain Technology Institute, Yalelaan, 44 Utrecht, The Netherlands

**Keywords:** Arteriovenous malformation, Pediatrics, Multimodality treatment, Functional outcomes, Development, Education

## Abstract

**Background:**

Arteriovenous malformations (AVMs) in the pediatric population are rare, yet they form the most frequent cause of hemorrhagic stroke in children. Compared to adults, children have been suggested to have beneficial neurological outcomes. However, few studies have focused on other variables than neurological outcomes. This study aims to assess the long-term functional and educational outcomes of children after multimodality approach of treatment for intracranial AVMs.

**Methods:**

All children treated in our center between 1998 and 2016 for intracranial AVMs were reviewed. Patient characteristics, as well as AVM specifics, were collected. Functional outcomes were compared using the modified Rankin scale (mRs). Educational levels, using the International Standard Classification of Education (ISCED), were compared to the age-matched general population of the Netherlands.

**Results:**

In total, 25 children were included at mean age of 10 years (range 2–16 years). Nineteen patients (76%) presented with intracranial bleeding. Mean follow-up was 11.5 ± 5.3 years (range 4.1–24.4). Four (16%) of patients were treated with embolization, three (12%) with microsurgery, and 18 patients (72%) received a combination of different treatment modalities. Altogether, 21 (84%) were embolized, 14 (56%) were treated with microsurgery, and eight (32%) received stereotactic radiosurgery. One child had a worse mRs at discharge compared to admission; all others improved (*n* = 11) or were stable (*n* = 13). At follow-up, all patients scored a stable or improved mRs compared to discharge, with 23 children (92%) scoring mRs 0 or 1. These 23 children followed regular education during follow-up without specialized or adapted schooling. No significant differences in educational level with the age-matched general population were found.

**Conclusion:**

This retrospective review shows positive long-term results of both functional and educational outcomes after multidisciplinary treatment of pediatric brain AVMs.

## Introduction

Arteriovenous malformations (AVMs) in the pediatric population are rare, yet they form the most frequent cause of hemorrhagic stroke in children [15]. Besides rupture and subsequent bleeding out of the AVM, children can also present with seizures and headaches [11].

Because not all AVMs are symptomatic, real incidence rates remain unclear. Prevalence of symptomatic AVMs is estimated to be between 0.06 and 0.11% in the total population [11], with mean age of diagnosis of 31.2 years [13]. Due to the longer lifespan, it is believed that children with AVMs would have a higher lifetime risk of bleeding than adults [10]. Furthermore, after treatment, re-bleeding occurs in 2.71% (SD ± 1.32%) of cases, which adds to the risk of this specific population [5]. Pathophysiology of genesis and rupture of AVMs is considered to be multifactorial [21]. Although congenital origin is considered an important factor, growing understanding on angiogenesis with theories on hormonal influences is also starting to become more accepted [21].

Upon bleeding, up to one third of children are comatose, and approximately half of patients have a neurological deficit [5]. The possible life threatening conditions, as a result, sometimes call for intensive care and emergency intervention. In general, a multidisciplinary approach of treatment is used to get the best possible results. Complete obliteration is considered an important purpose of treatment, as partial occlusion of AVMs is associated with re-bleeding. Possible modalities to reach this objective are embolization, microsurgery, and radiosurgery. These all have different advantages and disadvantages, and combinations could result in non-inferior outcomes [9, 17, 21]. However, due to the low incidence rates and lack of comparative trials, decision making on which combination is most appropriate remains largely experience- and expert-based.

Children have been suggested to have beneficial neurological outcomes [17], especially compared to adults [20]. Previous studies investigating cognitive outcomes after pediatric intracranial hemorrhage (ICH) used various questionnaires, as well as educational placement as outcome for cognitive function [1, 6, 12, 16]. Educational level and followed schooling might contain a natural and comprehensive overview of cognitive functioning as there is a strong relationship between these two [2, 8]. To our knowledge, no single study has investigated cognitive outcome specifically in children treated for intracranial AVMs. Therefore, the effects of AVMs, eventual bleeding, and treatment on intellectual capacity remain somewhat unclear. The present study aims to assess the cognitive function by educational outcomes of children after multimodality approach of treatment for intracranial AVMs.

## Methods

All children who underwent any form of treatment at our center for an intracranial AVM between 1998 and 2016 were reviewed. This specific timeframe was chosen so that a meaningful assessment of long-term follow-up could be done. Data were collected by using the digital medical records and by telephone call contact with patients or their parents. All patients who met the following criteria were included: 1) any form of treatment for intracranial AVM; 2) age below 18 at time of first treatment. No exclusion criteria were used.

### Variables

We collected patient age (years), gender, and presenting symptoms. Additionally, AVM size, localization, and the presence of deep venous drainage were obtained, and each AVM was graded following the Spetzler-Martin grading system (SMG) [22]. All treatment modalities used for each patient were reviewed, and we distinguished primary treatment and following treatments. Occlusion rates were obtained by trained radiologists as part of normal follow-up. The modified Rankin scale (mRs), adjusted for children, was used to score neurological disability [4]. At admission, discharge and in follow-up, mRs scores were collected in order to assess the efficacy of treatment. MRs higher than 2 was considered an unfavorable outcome. Additionally, the educational career of each patient was mapped out to assess cognitive functional outcome. We recognized severe neurological impairment and death as result of treatment as major complications.

### Educational grading

As a control group for highest achieved educational levels, we used the general population of the Netherlands, matched by age. This data was collected from a public database (Dutch Central Bureau for Statistics, accessed November 1, 2017) [7]. The educational system in the Netherlands can be divided roughly into primary, different levels of secondary, and tertiary education, following the International Standard Classification of Education (ISCED) 2011 [18]. However, ISCED level 4 is not present in the Dutch schooling system. Therefore, we chose to classify educational levels in lower, intermediate, and high education, in order to come to a meaningful comparison. Lower education hereby corresponds to primary and lower secondary education (ISCED level 1 and 2), intermediate education corresponding to upper secondary education (ISCED level 3), and high education to tertiary education (ISCED level 5 and 6).

### Statistical analysis

Statistical analysis was conducted using IBM SPSS statistics 24. To determine significant differences in the distribution of baseline characteristics we used the following tests: 1) Fisher tests for binary variables if the expected value was below five; 2) chi-squared tests if the expected value was five or higher; 3) and Student’s *t* test was used for all continuous variables. To reveal potential predictors, univariate analysis was done by using logistic regression. For all tests, *p* values below 0.05 were considered as statistical significant.

## Results

### Demographics of study population

In total, 25 children were included at mean age of 10 years (range 2–16 years). Demographics, Spetzler-Martin grades, and mRs at admission are summarized in Table 1. Nineteen patients (76%) presented with intracranial hemorrhage. The six patients with unruptured AVMs presented with headaches (*n* = 3), seizures (*n* = 1), and a defect in the skull (*n* = 1), and one patient was a coincidental finding. Four (16%) patients were treated in the acute setting on the same day as the hemorrhage. Mean follow-up was 11.5 years (range 4.1–24.4 years) (SD 5.3). Mean age at follow-up was 22.0 years (range 12.4–33.7 years) (SD 5.8).

**Table 1:** Patient demographics, mRs at admission, and radiological characteristics

	*N*	%	Test
Patients	25		
Age	10.5 years	SD 3.7	
Male	13	52%	*p* = 1.0
Presentation			
Bleeding	18	72%	
Headache	3	12%	
Seizure	2	8%	
Other	2	8%	
mRs presentation			
0	4	16%	
1	7	28%	
2	1	4%	
3	2	8%	
4	3	12%	
5	8	32%	
6	0	0%	
AVM characteristics			
Ruptured	19	76%	*p* = 0.016*
Size < 3 cm	11	44%	
Size 3–6 cm	12	48%	
Size > 6 cm	2	8%	
Infratentorial	5	20%	
Deep venous drainage	10	40%	
Associated aneurysm	3	13%	
Spetzler-Martin grade			
1	3	12%	
2	9	36%	
3	10	40%	
4	1	4%	
5	2	8%	

*mRs* modified Rankin scale, *AVM* arteriovenous malformation

*A *p* value < 0.05 was considered statistically significant

### Genetics

One patient was diagnosed with Wyburg-Mason syndrome. Another patient had unspecified congenital psychomotor impairment and diagnosed early in life with an accompanying AVM. Furthermore, two patients had family history of multiple family members with subarachnoidal hemorrhage or AVMs, yet without a syndromal diagnosis. Finally, one patient had accompanying Marfan syndrome.

### Treatment

Embolization, surgical resection, and stereotactic radiosurgery (SRS) using a linear accelerator or gamma-knife were used to treat the AVMs (Table 2). Embolization was the initial treatment in 20 (80%) patients, with the remaining five (20%) initially treated by microsurgical resection. SRS in this series was not used as initial treatment mode. In total, 21 (84%) patients underwent embolization, 14 (56%) were operated, and eight (32%) had subsequent SRS. The majority of the children (76%) were treated with multiple modalities. In three out of four cases of acute intervention, microsurgery was the primary treatment, and embolization was performed in the fourth. The two cases presenting with epilepsy were initially managed with anti-epileptic drugs. In both cases, the AVM as epileptic focus was thereafter treated by a combination of embolization and microsurgery. Afterwards, the seizures resolved in both patients, and anti-epileptic drugs were discontinued. Upon discharge, all patients would have access to rehabilitation support, which is part of the standard of care. The extent of this support differed between patients, according to the specific needs. The follow-up regimen consisted of angiographies to assess the extent of the occlusion, as well as outpatient control visits. In the first year post-treatment, cerebral angiography would take place yearly. Later on, the interval was extended, with a final angiography at the age of 21. Additional cerebrovascular imaging was performed if indicated.

**Table 2:** All used treatment combinations, grouped in unruptured and ruptured AVM status, with accompanying overall occlusion rate

Modalities	Total, *n*(%)	Unruptured, *n*(% total)	Ruptured, *n*(% of total)	Occlusion rate %
Embolization	4(16)	2(33)	2(11)	83
Embolization-Microsurgery	9(36)	2(33)	7(37)	100
Embolization-SRS	7(28)	1(17)	6(32)	90
Microsurgery	3(12)	1(17)	2(11)	98
Microsurgery-Embolization	1(4)	0	1(5)	100
Microsurgery-SRS	1(4)	0	1(5)	100
Total	25	6	19	94

*SRS* stereotactic radio surgery

### Outcome

Post-treatment angiography showed complete occlusion of the AVM in 19 of 25 patients (76%). The mean obliteration percentage of the total cohort was 93.4% (SD 15.5%). Acute treatment as well as the form of primary treatment was not associated with lower occlusion rates (both *p* = 1.00). However, AVMs with a SMG lower than 3 had higher occlusion rates (*p* < 0.001) and more often complete obliteration (*p* = 0.04). Upon treatment, two (8%) children experienced temporary increase of neurological deficits, and one (4%) had a new mild coordinative difficulty in one leg. Furthermore, three (12%) patients experienced post-treatment hydrocephalus. In two of these, this was due to the intracranial hemorrhage. In the third case, besides the patients preexisting psychomotor retardation, no other explanation was found. All patients with hydrocephalus needed an additional ventriculoperitoneal shunt. Re-bleeding occurred in three (12%) patients, of which two could be seen as AVM recurrence after initial complete AVM obliteration. All needed additional treatment. In the total cohort, no major complications or deaths occurred.

**Fig. 1:**
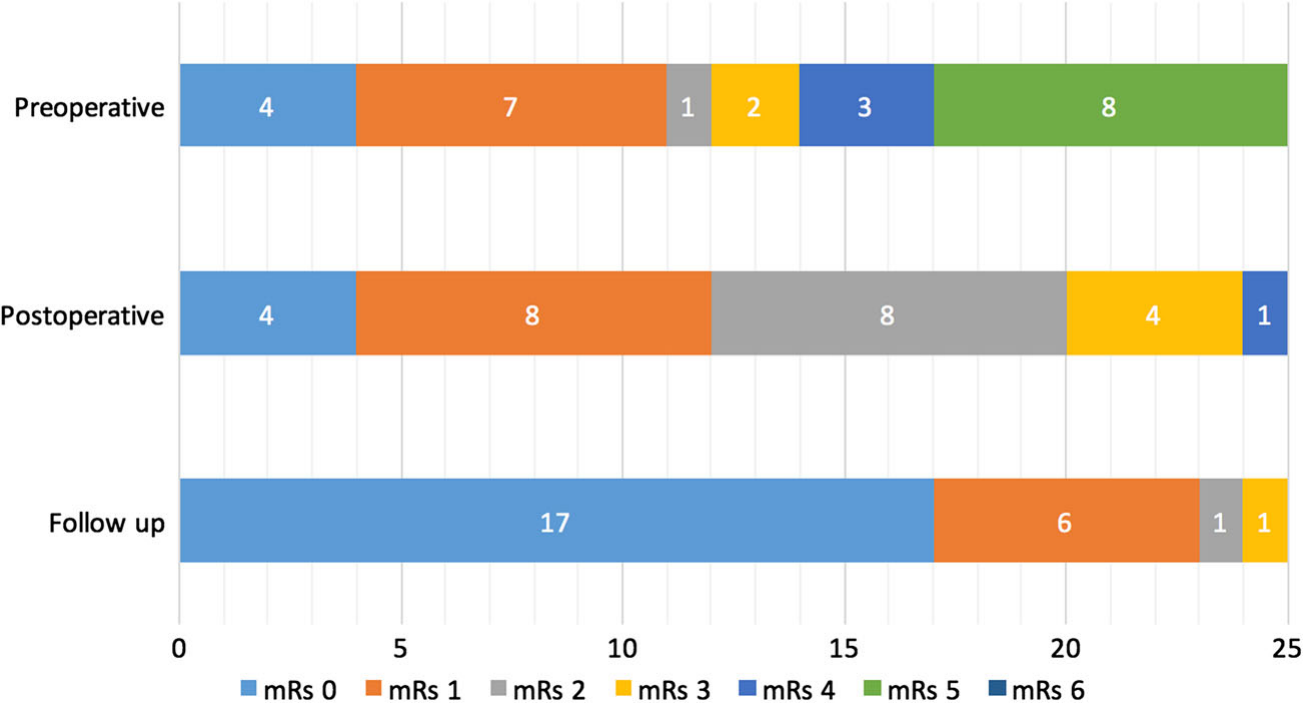
Modified Rankin scores at admission, at discharge, and in long-term follow-up. Numbers in the different boxes represent the corresponding amount of children with that specific mRs. Proportions of mRs scores at different moments in course of treatment and follow-up. mRs modified Rankin score. Admission represents mRs at admission. Discharge represents mRs at discharge. Follow-up represents mRs at the last moment of follow-up, average after 11.5 years

Comparing mRs at admission and at discharge, 52.6% of the children with a ruptured AVM improved (*n* = 10) and 5.3% (*n* = 1) scored worse. Of the patients with unruptured AVMs, 16% improved (*n* = 1) and none scored worse. At follow-up, five patients had persisting and stable neurological deficits, and three children had persisting but improved deficits. Six of these patients had mild deficits like visual field deficits (*n* = 5), diplopia (*n* = 1), and the previously mentioned coordinative difficulties (*n* = 1) with no interference in their daily life. In the other two patients, remaining deficits were hemiparesis (*n* = 1) and mental retardation (*n* = 1). The patient with mental retardation was known with these disabilities prior to treatment. Finally, in follow-up, all patients scored a stable or improved mRs compared to discharge. Of the children, 91.3% had either mRs 0 or 1, whereas the other two scored mRs 2 and mRs 3 respectively. The mRs scores at admission, discharge, and follow-up are arranged in Fig. 1.

### Education

**Fig. 2:**
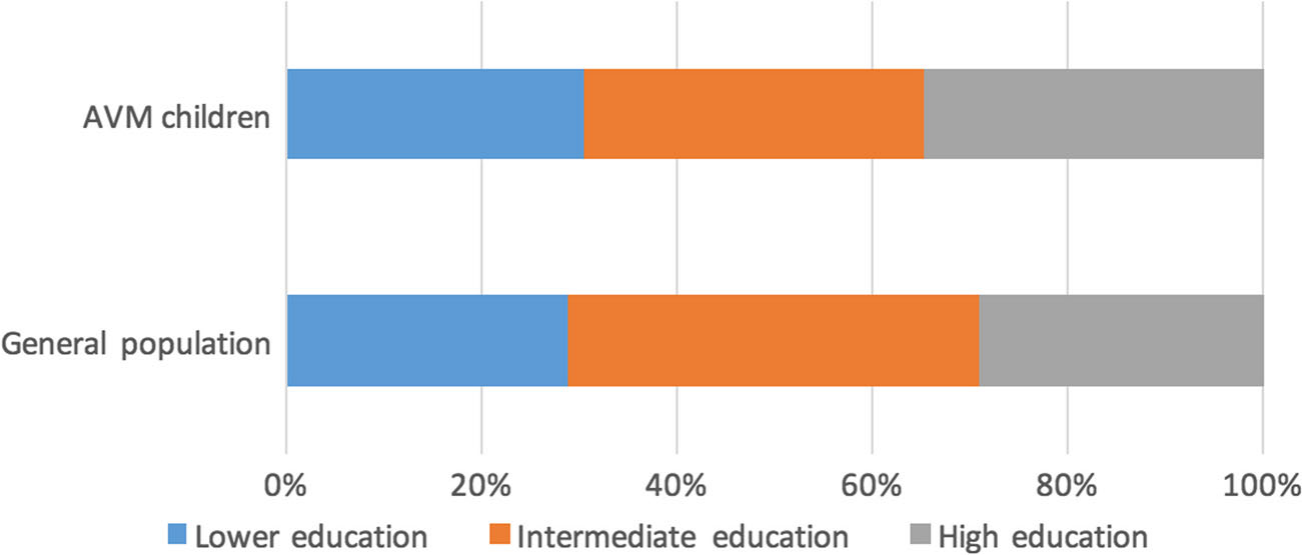
Distribution of educational levels of children treated for AVMs compared with the general population of the Netherlands. Proportions of educational levels. Lower education corresponding with ISCED levels 1 and 2, intermediate education corresponding with ISCED level 3, high education corresponding with ISCED levels 5 and 6. Educational levels of two children were not obtainable, resulting in educational levels of 23 children. The total general population of the Netherlands in the age-group 15–35 years is 4,178,000 persons.

During follow-up, 23 of the 25 children (92%) followed regular education without specialized or adapted schooling. One patient needed adjustments for physical disabilities with normal educational levels. Only one patient, who was known to have mental retardation previous to treatment, was in need of adaptive education. Furthermore, eight (34.8%) patients attained tertiary education, which we scored as high education. Of two children, specific educational levels were not obtainable, and only a simple assessment could be made. Therefore, we did not implicate these children in the comparison with the general population. The distribution of different educational levels of this case series in comparison to age-matched peers is shown in Fig 2. No differences were found.

### Statistical analysis

In this cohort, there was no significant relationship between unfavorable neurological or functional outcome (mRs > 2, found in two patients) and bleeding of the AVM, size (grouped following the SMG), or location (eloquent versus non-eloquent of the AVM, modified Rankin scale at admission, recurrence or re-beeding, and used treatment modalities (all *p* > 0.05). Although patients with SMG below 3 more often had complete obliteration, this association was not significant in logistic regression (*p* > 0.05). Reckoning the fact that re-bleeding only occurred in three cases, incomplete occlusion of the AVM was not associated with re-bleeding (*p* = 1).

## Discussion

This retrospective review shows positive long-term results of both functional and educational outcomes after a multidisciplinary approach of treatment in pediatric brain AVMs. This study investigated educational achievement, as this both reflects cognitive function and is particularly important for the quality of life of these patients. Almost all patients were able to continue their normal lives with minimal to no remaining neurological disabilities or cognitive restrictions. In comparison to age-matched peers, the educational levels of these children showed no difference.

Since the long-term functional outcome of the cohort was very good, it was impossible to find a predictor for unfavorable outcome. Because of the same reason and because less than a third of the patients were treated with a single strategy, no single treatment strategy was found superior in this case series. These results may confirm the importance of a multidisciplinary approach of this rather serious condition in the pediatric population. To our knowledge, there currently are no studies available comparing results of different treatment modalities and combinations. However, combining modalities is suggested only to improve clinical outcomes [9, 17]. A recent study (2016) on multimodality treatment of pediatric brain AVMs showed results very similar to this study, with the majority of pediatric patients scoring mRs between 0 and 2 at follow-up [17]. Innovation in different treatment options has been held accountable for a significant part of the improvement of results over the years [21]. A striking example is the decline in mortality rates. Early studies reported death following treatment in up to 25% [14]. However, most studies these days report mortality of 5% or even less [11, 14]. Adopting this trend, no children died in this study during admission or follow-up. Additional information about the amount of children who die at home or before intervention can take place would be useful to further determine outcome of children with AVM. Based on the administration of our hospital (region of 3.5 million people) combined with data of the Dutch Central Bureau of Statistics (CBS), a case of spontaneous death in a child because of an AVM rupture is highly unlikely to have occurred in our hospital in the past 19 years.

At presentation, prognosis may seem unclear to parents and clinicians. AVM bleeding can be life threatening and sometimes even requires emergency treatment. This study shows that in our cohort, neurological as well as educational outcomes are promising after multidisciplinary approach of treatment. Analysis of patient- and AVM-related factors did not result in significant associations with differences in outcomes. This is in contrast with some of the existing literature, in which associated aneurysms, deep venous drainage, and partial occlusion are suggested to be associated with higher chance of re-bleeding and worse outcomes [5]. Furthermore, comatose condition at presentation has been associated with less favorable outcomes [5]. However, these correlations were not found in this study; although eight patients (34.8%) had a mRs of 5 at admission, seven of these (87.5%) had mRs 0 in follow-up, and all followed normal education. Moreover, the distribution of educational levels among all treated children gives the impression that the learning abilities have also not been affected. In total, eight patients (34.8%) achieved high education with bachelor’s and even master’s degrees. This is more or less equal to 29.1% in the general population. These results are noteworthy, as some studies on pediatric ICH note significantly lower cognitive as well as educational function in follow-up [11, 15]. It has also been suggested that over half of these children need additional educational services beyond 12 months after ICH [12]. However, these findings cannot be supported by our case series, where only 8% needed additional educational services, and education seemed not to be affected. It has also been described that the need of specialized education and corrective devices decreases health-related quality of life (HRQL) in these children [1]. As education and schooling make up a considerable part of children’s life, the ability to follow regular schooling is very important. This underlines the significance of these favorable results.

Due to the low incidence rates, true evaluation of the effects of any form of treatment and neurological and cognitive outcomes remains difficult. With 25 patients in as many years and multiple options for treatment, we still depend largely on retrospective analyses, which is also the main limitation of this study. The fact that some of the previously described factors related to changes in outcomes were not found significantly associated, might very well be based on the small size of this cohort. Presumably, a larger population may have demonstrated a wider range of outcomes. In addition, the use of a subjective scoring system should also be noted as a possible bias. The modified Rankin scale is a validated adult scoring system and commonly used in clinical trials [3, 23]. Although several previous studies have used an adjusted modified Rankin scale for children, this scoring system has yet to be validated in the pediatric population [4]. Additionally, the subjective nature of the scale holds possible reporting bias [3, 19]. Furthermore, by evaluating educational levels, we did not differentiate between different aspects of cognitive functioning. For this kind of evaluation, a validated measurement of cognitive function should have been used. Therefore, we would like to emphasize that these educational outcomes should not directly be translated to cognitive outcomes. Although, as described earlier, there is a strong relation between the two, they are not equivalent. Therefore, results should be interpreted with consideration of these limitations and remarks. These outcomes should also be translated only to countries with comparable standard of care and rehabilitative services, with similar accessibility. The fact that two out of three patients with a re-bleeding had initial complete obliteration demonstrates the importance of post-treatment follow-up and angiographies.

## Conclusion

This retrospective review shows satisfactory long-term results of both functional and educational outcomes after multidisciplinary treatment of pediatric brain AVMs. Unfortunately, it was impossible to find predictors for either better or worse outcomes. However, these findings might contribute to the existing belief that clinical outcomes seem to be favorable among children as compared to adults.
